# Introducing *Oxford Open Neuroscience*

**DOI:** 10.1093/oons/kvac005

**Published:** 2022-05-04

**Authors:** Sam Gilbert, Carlos Ibáñez, Alicia Izquierdo, Orly Reiner, Hongyan Wang

**Affiliations:** 1 Institute of Cognitive Neuroscience, University College of London, London, UK; 2 McGovern Institute for Brain Research, School of Life Sciences, Peking University, Beijing, China; 3 Department of Psychology, University of California Los Angeles, Los Angeles, CA, USA; 4 Department of Molecular Genetics and Molecular Neuroscience, Rehovot, Israel; 5 Neuroscience & Behavioral Disorders Programme, Duke-NUS Medical School, Singapore

## INTRODUCING *OXFORD OPEN NEUROSCIENCE*

Welcome to *Oxford Open Neuroscience*! We are excited to introduce this new open access journal, which provides a venue for top quality papers covering the widest interpretation of neuroscience, including cellular and molecular neuroscience, behavioral neuroscience, developmental neuroscience, disease neuroscience and cognitive neuroscience.

The scope of *Oxford Open Neuroscience* includes research into the nervous system in both health and disease, encompassing basic, translational and clinical research. Interdisciplinary work and technological advancement are also welcomed. The journal publishes scientific research of the highest quality, rigor, and reproducibility, including reviews, registered reports, commentaries and debates. In addition to original research, *Oxford Open Neuroscience* welcomes theoretical, technical and methodological papers within any field of neuroscience. *Oxford Open Neuroscience* aims to represent the entire neuroscience community and supports diversity and fairness in the editorial process.

## EDITORIAL STRUCTURE

*Oxford Open Neuroscience* is run by a representative group of active scientists who are subject specialists, rather than a single editor-in-chief. Representing the needs of that community and making science-based decisions, the journal’s senior editors act as ambassadors for their individual fields. Key decisions around the journal’s development, strategy and policies are made collectively and in consultation with the Editorial Board. *Oxford Open Neuroscience*’s senior editors are the following:

- Sam Gilbert, *Cognitive Neuroscience*
- Carlos Ibáñez, *Cellular and Molecular Neuroscience*
- Alicia Izquierdo, *Behavioral Neuroscience*
- Orly Reiner, *Disease Neuroscience*
- Hongyan Wang, *Developmental Neuroscience*

This structure roots a truly researcher-focused ethos in the foundation of the journal while still concentrated on publishing excellent research conducted with high scientific rigor.

## EDITORIAL INNOVATIONS

*Oxford Open Neuroscience* encourages submissions from all neuroscientists, regardless of position, affiliation or country of origin, and encourages debate and discussion in the journal through new article types.

The journal also seeks to address some of the larger problems in research publishing through transparent approaches to the peer review process. *Oxford Open Neuroscience*

- minimizes bias by operating double blind peer review;
- emphasizes transparency through the authors’ receipt of editor summaries, which adjudicate reviewers’ comments where necessary and clarify requirements for publication;
- emphasizes transparency through the publication of anonymous reviewer comments alongside the published manuscript; and
- maximizes review speeds and publication efficiency by offering authors a ‘no-revision’ option.

Those interested can read more about these new approaches here.

## OPEN ACCESS

*Oxford Open Neuroscience* is a high-quality, fully open access, funder-compliant venue for neuroscience authors. As an open access journal, it offers authors a high-visibility platform and broad readership for their research to make maximum impact. It will continue to support other open research and open data practices as they emerge.

With the focus of fairness, equity and diversity in the forefront, Oxford University Press (OUP) intends that no authors will be barred from publishing in the journal from a lack of funding. OUP offers several routes to publishing in the journal, including accessible open access fees, an expanding number of Read and Publish agreements that cover publication in open access journals for affiliated authors, publication waivers under its Developing Countries initiative and discretionary open access fee waivers.

### CONCLUDING REMARKS

As a researcher-led publication with a focus on diversity, transparency and innovation, our hope is that *Oxford Open Neuroscience* will provide a fully open access alternative to more traditional neuroscience journals and propel the field forward into a new era of research publishing.

Finally, we would like to express many thanks for the contributions made by recent and future authors, reviewers, Editorial Board members and associate editors, as well as the publishing team at OUP. Most importantly, we want to thank you, the reader of this first set of publications and hopefully of publications to come. Once again, welcome to *Oxford Open Neuroscience*!

Sincerely,

Sam Gilbert, Senior Editor–Cognitive Neuroscience, UK

Carlos Ibáñez, Senior Editor–Cellular and Molecular Neuroscience, China

Alicia Izquierdo, Senior Editor–Behavioral Neuroscience, USA

Orly Reiner, Senior Editor–Disease Neuroscience, Israel

Hongyan Wang, Senior Editor–Developmental Neuroscience, Singapore

